# Modeling of hydrogen atom diffusion and response behavior of hydrogen sensors in Pd–Y alloy nanofilm

**DOI:** 10.1038/srep37043

**Published:** 2016-11-15

**Authors:** Yi Liu, Yanli Li, Pengcheng Huang, Han Song, Gang Zhang

**Affiliations:** 1 School of Mechanical and Electronic Engineering, Wuhan University of Technology, Wuhan 430070, China; 2 School of Mechanical Science and Engineering, Huazhong University of Science and Technology, Wuhan, 430074, China

## Abstract

To detect hydrogen gas leakage rapidly, many types of hydrogen sensors containing palladium alloy film have been proposed and fabricated to date. However, the mechanisms and factors that determine the response rate of such hydrogen sensor have not been established theoretically. The manners in which response time is forecasted and sensitive film is designed are key issues in developing hydrogen sensors with nanometer film. In this paper, a unilateral diffusion model of hydrogen atoms in Pd alloy based on Fick’s second law is proposed to describe the Pd–H reaction process. Model simulation shows that the hydrogen sensor response time with Pd alloy film is dominated by two factors (film thickness and hydrogen diffusion coefficient). Finally, a series of response rate experiments with varying thicknesses of Pd–Y (yttrium) alloy film are implemented to verify model validity. Our proposed model can help researchers in the precise optimization of film thickness to realize a simultaneously speedy and sensitive hydrogen sensor. This study also aids in evaluating the influence of manufacturing errors on performances and comparing the performances of sensors with different thicknesses.

Hydrogen gas is a widely used raw material and product (or byproduct) of modern industries^1^. However, the gas is flammable and easily explodes in the air under normal conditions at concentrations ranging between 4.65% and 74.5%^2^. Hence, almost all production activities involving hydrogen gas entail extremely safe monitoring systems to prevent any leakage.

In general, the main research interest in hydrogen leakage detection is how to reduce the time between the hydrogen leak and the corresponding alarm and to increase the chance of escaping the dangerous site or shutting down the device^3^,^4^,^5^,^6^. Optical fiber hydrogen sensors play a highly important role in monitoring hydrogen leakage because of their anti-electromagnetic interference and intrinsic safety ability. Most optical fiber hydrogen sensors employ palladium as the transducer element because of the intrinsically high sensitivity and selectivity toward hydrogen^7^,^8^. To improve the sensor response rate, numerous experimental prototypes for hydrogen gas detection have been developed^9^,^10^,^11^,^12^. Zhao *et al*. found that response time is strongly dependent on the α, mixed α/*β*, and *β* Pd–hydride phases formed in the films. The longest response time (about thousands of seconds) occurred at the hydrogen concentration corresponding to the α → *β* phase transition region. The phase transition region can be adjusted by changing the content of the alloy element^13^. Song *et al*. proposed a kind of annealing-stimulated method to retard the aging behavior of thin film and to improve the response speed^14^. Kay *et al*. studied the kinetics of hydrogen absorption by bulk Pd (110); they further demonstrated that in the α-phase region, where concentration is low, the hydrogen absorption rate of Pd is limited by the diffusion process of hydrogen atoms in the bulk rather than the chemisorption of H_2_ molecules on the Pd surface. The research results well demonstrated the dominant factors of penetration time in bulk Pd. However, such results are difficult to be used in forecasting the response time of Pd nanofilm, particularly, the single surface of the film exposed to hydrogen^15^.

These experiments indicate that the dimension and content of Pd nanometer film play the most important roles in rapid hydrogen detection. However, few theoretical analyses have been performed on the diffusion behavior and response time of Pd or Pd alloy thin films exposed to hydrogen. For a different application, precise designing the Pd or Pd alloy thin film to satisfy the measuring requirements is also a crucial issue. In this paper, a hydrogen single-side diffusion model of the Pd-based metal thin film was established, and a function was derived to describe the influences of the dimension and content of the thin film on the response time. Specifically, we have performed experiments on the optical reflectance response characteristics of Pd–Y films to validate the diffusion model. We theoretically and experimentally confirmed that the response time holds a direct ratio to the square of the film thickness. This conclusion benefits the precise designing of nanofilm thickness to satisfy the different requirements for response rate.

## Hydrogen Diffusion Model in Pd-based Metal Thin Films

### Hydrogen diffusion model based on Fick’s second law

When hydrogen molecules meet Pd, the former dissociate into hydrogen atoms on the Pd film surface. The hydrogen atoms then dissolve and diffuse in Pd and form an interstitial solid solution PdH_x_ (*x* is the atomic ratio of H/Pd). When the hydrogen concentration decreases, the hydrogen atom desorbs from the Pd film surface. This reaction is reversible. Given that the hydrogen concentration in the film evolves, the diffusion of hydrogen atoms is a typical non-steady state diffusion process that can be described by Fick’s second law^16^.

**Figure 1:**
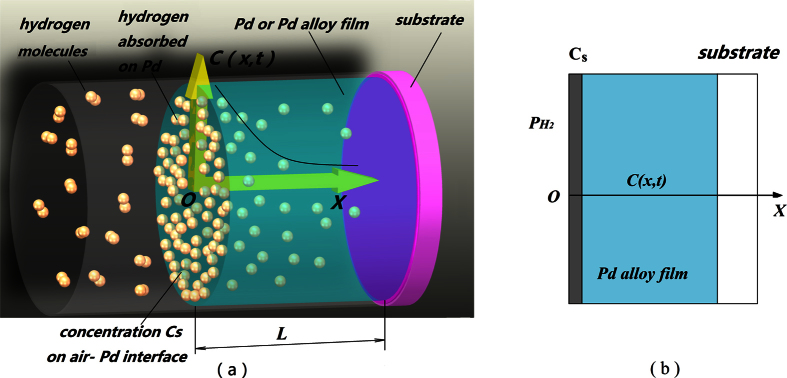
Hydrogen atom diffusion in the Pd-based alloy film; (**a**) Diffusion process; (**b**) Coordinate system of the model based on Fick’s second law.

The Pd-based thin film in the optical fiber hydrogen sensor is usually coated at the end of the optical fiber (or on one side of a substrate) and exposed to the hydrogen gas. The Pd–H_2_ interaction model is shown in Fig. 1(a). This model consists of hydrogen dissociating on the outside surface, diffusing in the inner Pd film, and restraining on the Pd–substrate interface. Generally speaking, the substrate that supports the Pd-based thin film cannot be penetrated by hydrogen. Therefore, it is a diffusion-limited reaction in one dimension with a constant source in solid. A coordinate system O is established at the center of the interface between the film and the hydrogen gas [Fig. 1(b)]. The x-axis is perpendicular to the film surface. We denote the thickness of the thin film as *L*, the diffusion coefficient of the hydrogen atom in the Pd-based metal nanofilm as *D*, and the hydrogen atom concentration as *C*(*x, t*), which is a function of the time *t* and the position *x* in the Pd-based metal nanofilm. When the film comes in contact with the hydrogen gas (H_2_ partial pressure is P_H2_), the hydrogen molecule is dissociated continuously into hydrogen atoms on the solid–gas interface, and a constant concentration of hydrogen atoms C_s_ is formed on the interface. According to Fick’s second law, the hydrogen atom concentration *C*(*x, t*) satisfies a second-order partial differential equation (PDE) as follows:


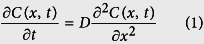


The hydrogen concentration in the film is zero before the Pd–H_2_ reaction; hence, the initial conditions of the equation are given as follows:


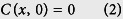


Furthermore, the hydrogen concentration on the solid–gas interface is a constant C_s_ determined by the hydrogen concentration in the surrounding environment. Thus, the first boundary condition is given as follows:


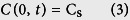


The hydrogen atoms diffuse in the opposite direction when they reach the film–substrate interface. This process is similar to the reflection of a flat mirror. The concentration gradient of the incident hydrogen atoms is equal to that of the reflected hydrogen. Therefore, the second boundary condition is given as follows:


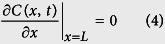


The PDE (1) is solved by using separation of variables method (also known as the Fourier method). The hydrogen atom concentration *C*(*x, t*) in the Pd-based metal film is obtained as follows:


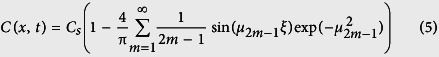


where 
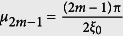
, 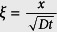, 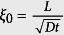.

Equation (5) shows the concentration distribution of hydrogen atoms at any time. When we design an optical fiber hydrogen sensor, whether the film is coated on the end or on the side of optical fiber, we detect the average effect of change on refraction index in the film. Therefore, the average concentration of hydrogen atoms in the film along the x-axis direction is given as follows:


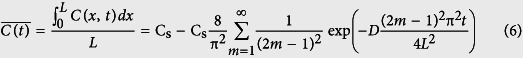


The average concentration of hydrogen atoms in the film 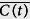 only depends on the time and film thickness and not on the film position. If we define the percentage of response as *η* to represent the completion level of reaction, the expression is given by:


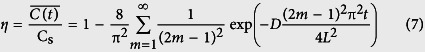


### Model discussion and simplification

#### (A) Transient concentration analysis and simplification

Equation (5) shows the transient concentration distribution of hydrogen atoms in the film at any time. The right-hand side of equation (5) is expanded as follows:


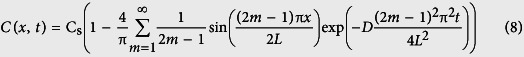


When time *t* is equal to zero in equation (8), the exponent term 
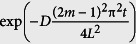
 corresponds to 1. Equation (8) is simplified as follows:


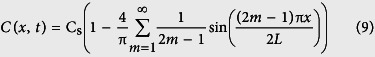


The infinite series in equation (9) is summed, and the details of derivation are included in Appendix I. Equation (9) can be rewritten as follows:


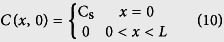


Equation (10) indicates that the hydrogen concentration is C_s_ at the gas–solid interface and 0 in the Pd-based metal film when time *t* *=* *0*. This representation means that the hydrogen atom diffusion in the film has not yet begun.

For *t* *>* *0*, the exponent term 
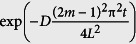
 attenuates rapidly in equation (8), particularly, in the high-order harmonic wave. If we choose the first two terms of the infinite series as the approximation of hydrogen concentrate distribution, the result is given by


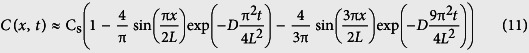


Moreover, the error of truncation is given by


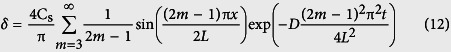


**Figure 2:**
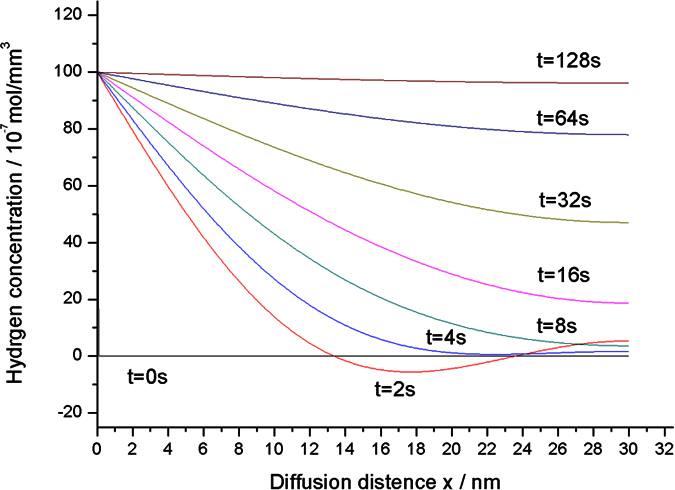
Concentration distribution of hydrogen in the Pd–Y alloy film.

A series of time, *t* *=* *0, 2, 4, 6, 8, 10, 20, 40* s, is proposed to study the concentration distribution of transient hydrogen in Pd–Y alloy film. We then suppose the thickness *L* of Pd–Y alloy film as 30 nm, diffusion coefficient *D* as 10 nm^2^/s, and the hydrogen concentration on the solid–gas interface C_s_ as 10^−5^ mol/mm^3^. The concentration distribution of hydrogen based on equations (10) and (11) is shown in Fig. 2. The hydrogen concentration in the film on the solid–gas interface is higher than that in the film. As time progresses, the hydrogen concentration increases. For *t* = 2, the curve is under y-axis, which is caused by the error of truncation.

#### (B) Average concentration analysis and simplification

With the same consideration, the first two terms of the infinite series in equation (6) are reserved as the approximation of the average hydrogen atom concentration in the film. The result is given by


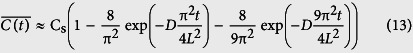


Meanwhile, the error of truncation is as follows:


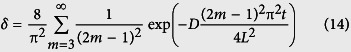


#### (C) Percentage of response simplification and analysis

On the basis of equation (13), the percentage of response *η* is rewritten correspondingly as


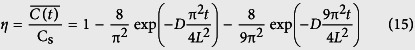


**Figure 3:**
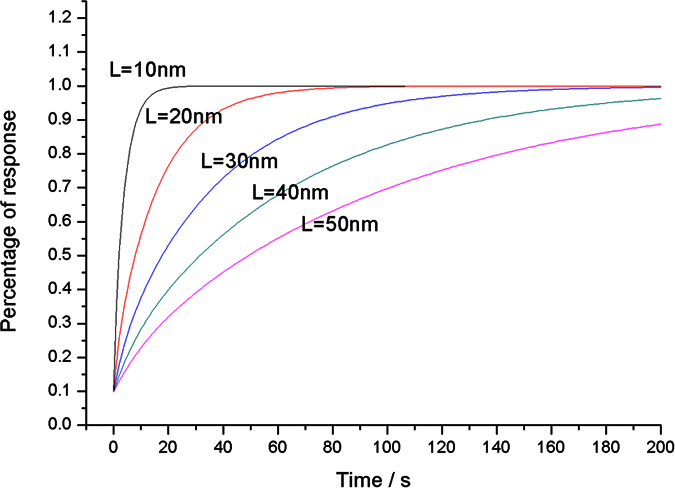
Percentage of response versus time with different film thicknesses.

Varying thicknesses, *L* = 10, 20, 30, 40, 50 nm, is proposed to study the influence of thickness on the response. We suppose the diffusion coefficient *D* as 10 nm^2^/s. The percentage of response based on equation (15) is displayed in Fig. 3. The figure illustrates that the percentage of response rises exponentially and that the thinner film can complete the reaction earlier.

#### (D) Response rate analysis

To determine the change rate in the percentage of response, we denote the response rate *V*_r_ as


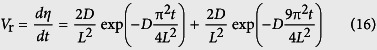


Equation (16) indicates that the sensor response rate is not a constant. The response rate is the highest at the initial status of the reaction and attenuates rapidly afterward. When time *t* approaches infinity, the response rate approaches 0, and the reaction proceeds toward the equilibrium status.

#### (E) Response time analysis

The percentage of response *η* is an exponent function of time *t*. Only when the time *t* approaches infinity can the percentage of response reach 100% and the hydrogen sensor reading can be stable. In practice measurement, we often regard the 90% of reaction as the stable value of reading. The response time *T*_response_ is defined as the time spent from reaction initiation to 90% steady state. Therefore, the response time must satisfy equation (17).


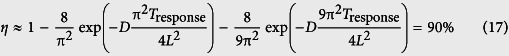


The typical value of the response time *T*_response_ changes within several seconds to tens of minutes. Thus, the second-order term in equation (17) is much smaller than the first-order term. Equation (17) can be approximated with the first-order term as follows:


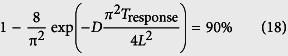


By solving equation (18), we can obtain response time *T*_response_ as follows:


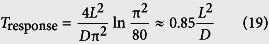


Equation (19) indicates that the response time *T*_response_ of hydrogen sensors based on Pd or Pd alloy film depends on the film thickness and diffusion coefficient. Decreasing film thickness and increasing diffusion coefficient can reduce response time and detect hydrogen gas leak rapidly.

## Experiments

### Experimental setup and principle

**Figure 4:**
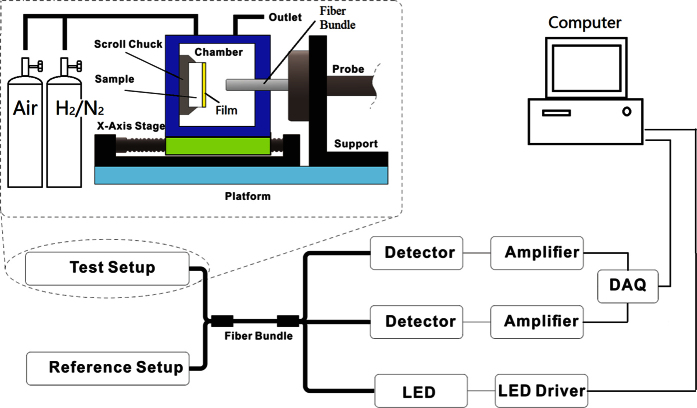
Optical measurement system of the hydrogen response.

In this work, a reflective optical fiber bundle sensor structure with compensation is used to record the reaction process of several Pd alloy films with different thicknesses when they load hydrogen. Figure 4 presents the diagram of the gas sensor system. Broadband light generated by a high-power LED is coupled into an optical fiber bundle. The light is split in two beams, namely, testing and reference signals, by the fiber bundle and then propagated to two chambers separately. The reflected signals from the two chambers are collected by their respective silicon photodiode. The signal originating from the reference chamber is only affected by the light source fluctuation. The final output *S* is only determined by the refractive index on the Pd alloy film surface after the errors are compensated by the reference signal.

Hydrogen exposure reduces both the real and imaginary parts of the Pd complex refractive index, which then increases output *S*^17^. *S* is a function of the average hydrogen concentration in the film. If we denote the initial value of *S* before the film is exposed to the hydrogen gas as *S*_0_ and the stable status value of *S* as *S*_∞_, the percentage of optical response of thin film *η*_e_ can be defined as


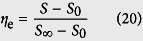


The percentage of optical response of thin film *η*_e_ increases gradually from 0 to 1 during the Pd–H reaction. If we record time *T*_e_ spent during the ascent processing of parameter *η*_e_, the time *T*_e_ corresponds to the response time of the film *T*_response_ in equation (19).

### Film preparation and characterization

**Figure 5:**
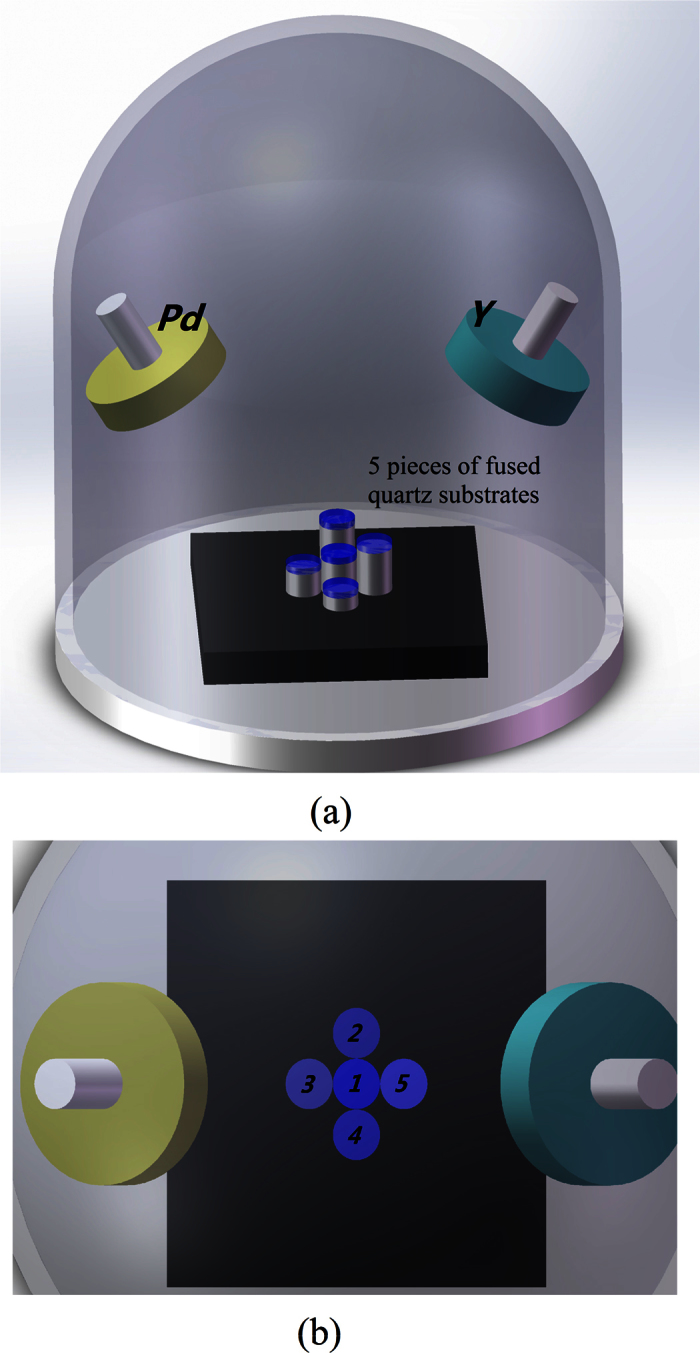
Five film pieces deposited simultaneously in the sputtering system. (**a**) All substrates are placed at the center of the sample platform in different layer heights. (**b**) The five films are assigned with ID from 1 to 5.

At one time, sputtering all films guarantees the uniformity of material composition and reduces the influence of the non-uniform diffusion coefficients on response time. Several similar substrates were placed in the sputtering chamber. These substrates were placed at the center of the sample platform and with different layer heights in the BESTECH sputtering system. Three-inch Pd and Y targets were installed in the DC and RF sources of the sputtering system, respectively. Under a sputtering pressure of Ar at 0.5 Pa, the deposition power was controlled to 100 W for Pd and 150 W for Y, which corresponded to the deposition rates of 1.3 and 0.2 Å/s, respectively, for the center substrate. The sputtering time was approximately 200 s. The sputtering parameters of the film deposited on the central fused quartz substrate (ID: #1) was monitored by a quartz crystal thickness monitor (Fig. 5).

**Figure 6:**
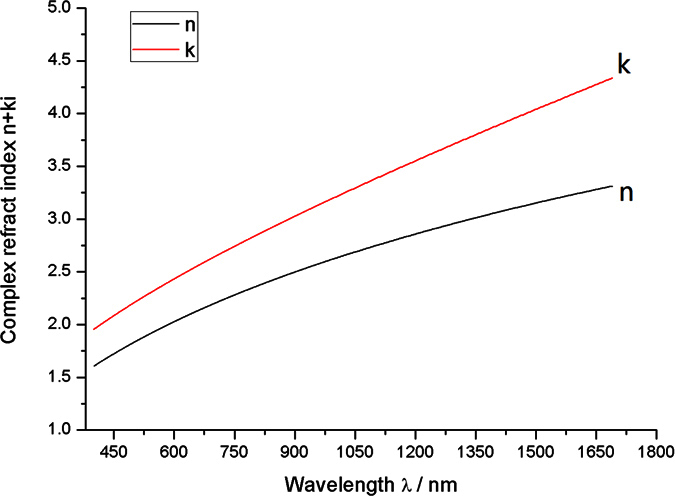
Refraction index of the Pd_0.92_–Y_0.08_ thin film.

Ellipsometry is a noncontact optical measurement technique that can obtain the thickness and dielectric properties of thin films by analyzing the change in polarization state between the incident and the reflect light on the thin film. An ellipsometry apparatus (M-2000 type produced by J. A. Woollam) was used to measure the thicknesses of the films prepared by the sputtering system. The thicknesses were 11.96 (ID: #4), 15.04 (ID: #3), 21.01 (ID: #5), and 29.84 nm (ID: #2). The #1 film, which was a calibration sample, was not used in the follow-up experiments. Meanwhile, the refractive index of the metal material is a complex number 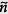, which consists of the real (index *n*) and imaginary parts (extinction coefficient *k*). The test results indicate the extremely close complex refracted indices of the five film pieces and the high consistency of the material composition of these films. The refraction index of the Pd_0.92_ − Y_0.08_ thin film is presented in Fig. 6.

## Results and Discussions

**Figure 7:**
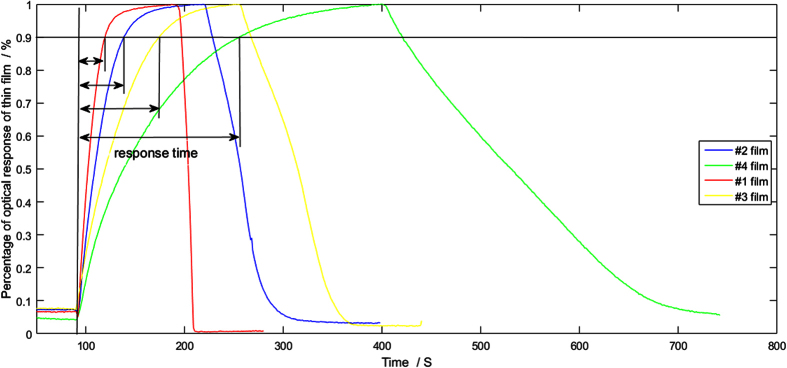
Optical response processing of the four pieces of Pd alloy thin films exposed to a mixture of 4% H_2_ in N_2_. Vaious response times are obtained for different thicknesses.

The four pieces of thin films are exposed to a mixture of 4% H_2_ in N_2_, respectively, to observe the response processing. Figure 7 shows the percentage of optical response of the four thin films. The curves shown in Fig. 7 are identical to those analyzed in Fig. 3, which all exhibit exponential growth characteristics. No overshoot is produced in the whole growth. When the time trends to infinity, the optical response of the thin film trends to 1. The thick film needs a much longer response time than that required by the thin film.

The four pieces of thin films are exposed to a mixture of 4%, 2%, and 0.5% H_2_ in N_2_, respectively. The response time is shown in Table 1.

**Figure 8:**
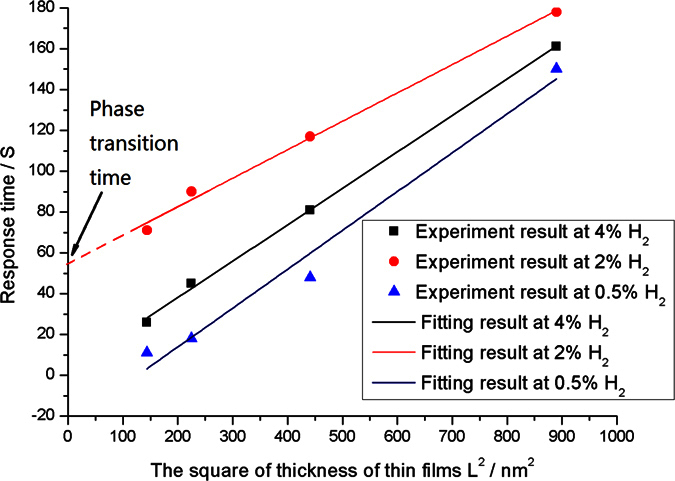
Relationship between the response time *T* and square of thin film thickness *L*^*2*^.

To clearly show the relationship between the response time *T* and the thickness of thin films *L*, a group of curves are displayed in Fig. 8. The horizontal coordinate represents the square of the film thickness *L*^*2*^. The vertical coordinate represents the response time *T*. At different hydrogen gas concentrations, the response time is in direct ratio to the square of film thickness. This aspect is identical to the theoretical analysis results (equation 19). The slope of the line is determined by the diffusion coefficient of the hydrogen atoms in Pd. Different reaction productions (α-PdH and β-PdH) are known at different hydrogen concentrations, leading to different paths of diffusion. In the experiment, three lines are very close to being parallel. This similarity demonstrates the similar diffusion coefficients for different diffusion patterns in the Pd–Y alloy film. We also found that the response time at 2% H_2_ is much longer than those at higher or lower concentrations of hydrogen gas (Fig. 8), which may be caused by the phase transition of PdH. In the phase transition concentration, the Pd material undergoes phase transition from α-PdH to β-PdH, which consumes a specific time. Hence, the response time at 2% H_2_ of each thin film is a specific time longer than those at other concentrations. The intercept on the y-axis of the line represents the specific time of phase transition. The phase transition time is about 50 s (Fig. 8). The slow response phenomena of sensors with Pd–Y alloy films induced by phase transition are also observed in Pd–Au alloy^13^.

## Conclusion

The mechanism and factors that determine the response rate of hydrogen sensors are key issues in the design and development of hydrogen sensors for the rapid leak detection. The response process of the sensor to the hydrogen gas is a chemical reaction, which determines the response rate. To describe this process theoretically, a unilateral diffusion model of hydrogen atoms in Pd alloy based on Fick’s second law is proposed. By resolving the diffusion model, we found that the sensor response is an exponential function as time changes. The response time of hydrogen sensor with the Pd alloy film is dominated by two factors (film thickness and hydrogen diffusion coefficient in Pd). The response time of the hydrogen sensor depends on the square of alloy film thickness. Experimental results not only validate this point, they also show the presence of close diffusion coefficients in the α-PdH and β-PdH. Our proposed model can be used to design the film thickness in hydrogen sensors for various rate requirements. The experiments also reveal the phase transition process and corresponding duration excluded in the diffusion model. Among the important issues for future investigation include which factors determine the phase transition time and how to reduce such duration.

## Additional Information

**How to cite this article**: Liu, Y. *et al*. Modeling of hydrogen atom diffusion and response behavior of hydrogen sensors in Pd–Y alloy nanofilm. *Sci. Rep.* **6**, 37043; doi: 10.1038/srep37043 (2016).

**Publisher’s note:** Springer Nature remains neutral with regard to jurisdictional claims in published maps and institutional affiliations.

## Supplementary Material

Supplementary Information

## Figures and Tables

**Table 1:** Response time of films at different hydrogen concentrations.

	Film thickness (unit: nm)	Response time in 4% H_2_ in N_2_ (unit: S)	Response time in 2% H_2_ in N_2_ (unit: S)	Response time in 0.5% H_2_ in N_2_ (unit: S)
ID: #4	11.96	26	71	11
ID: #3	15.04	45	90	18
ID: #5	21.01	81	117	48
ID: #2	29.84	161	178	150
