# Supplementary material for: Modeling of hydrogen atom diffusion and response behavior of hydrogen sensors in Pd–Y alloy nanofilm
Source: Sci Rep. 2016 Nov 15;6:37043. doi: 10.1038/srep37043 (PMC5109211; doi:10.1038/srep37043)
Supplement: Supplementary Information [file srep37043-s1.doc]

Modeling of hydrogen atom diffusion and response behavior of hydrogen sensors in Pd–Y alloy nanofilm

Yi Liu^a^ *, Yanli Li^a^, Pengcheng Huang^b^, Han Song^b^ , Gang Zhang^b^

*a School of Mechanical and Electronic Engineering, Wuhan University of Technology, Wuhan 430070, China*

*b School of Mechanical Science and Engineering, Huazhong University of Science and Technology, Wuhan, 430074,China*

**Appendix I**

1. When *x=*0, submitting *x=*0 into Eq. (9), the result is

$C(0,0)=\text{C}_{\text{s}}$ (a)

1. When *0<*x*<L*, the sum of series at the right hand of the Eq. (9) can be calculated as follows:

If suppose

$z=e^{\frac{\text{π}x}{2L}i}$ (b)

we have

$$\sum_{m=1}^{\infty} \frac{1}{2m-1}\cdot z^{2m-1}=\sum_{m=1}^{\infty} \frac{1}{2m-1}e^{\frac{(2m-1)\text{π}x}{2L}i}=\sum_{m=1}^{\infty} \frac{1}{2m-1}\cos\frac{(2m-1)\text{π}x}{2L}+i\sum_{m=1}^{\infty} \frac{1}{2m-1}\sin\frac{(2m-1)\text{π}x}{2L}$$

(c)

Notice that the image part of sum of series in Eq. (c)

$image(\sum_{m=1}^{\infty} \frac{1}{2m-1}\cdot z^{2m-1})=\sum_{m=1}^{\infty} \frac{1}{2m-1}\sin\frac{(2m-1)\text{π}x}{2L}$ is the series in Eq. (9). Hence, we can get the sum of series by summing the $\sum_{m=1}^{\infty} \frac{1}{2m-1}\cdot z^{2m-1}$ and then calculating its image part.

According to the Eq. (4.6.33) in [A1], the sum of series

$\sum_{m=1}^{\infty} \frac{1}{2m-1}\cdot z^{2m-1}=z+\frac{1}{3}z^{3}+\frac{1}{5}z^{5}+\cdot\cdot\cdot=\text{arctanh}(z)$ (d)

According to the Eq. (4.6.16) in [18], for a complex number $z=a+bi=e^{\frac{\text{π}x}{2L}i}$, the inverse hyperbolic function

$\text{arctanh}(z)=-i\arctan(iz)=-i(\frac{1}{2}\arctan(\frac{2a}{1-a^{2}-b^{2}})+\frac{i}{4}\ln[\frac{a^{2}+(b+1)^{2}}{a^{2}+(b-1)^{2}}])$ (e)

Considering Eq. (c) and submitting Eq. (e) into (d), we have

$\sum_{m=1}^{\infty} \frac{1}{2m-1}\sin\frac{(2m-1)\text{π}x}{2L}=image(\sum_{m=1}^{\infty} \frac{1}{2m-1}\cdot z^{2m-1})=image(\text{arctanh}(z))=-\frac{1}{2}\arctan(\frac{2a}{1-a^{2}-b^{2}})$(f)

Notice that $z=a+bi=e^{\frac{\text{π}x}{2L}i}$. The modulus of complex z is equal to

$\sqrt{a^{2}+b^{2}}=1$ (g)

Submitting Eq.(g) into Eq.(f), we have

$\sum_{m=1}^{\infty} \frac{1}{2m-1}\sin\frac{(2m-1)\text{π}x}{2L}=-\frac{1}{2}\arctan(\frac{2a}{1-a^{2}-b^{2}})=-\frac{1}{2}\cdot\frac{\text{π}}{2}=-\frac{\text{π}}{4}$ (h)

Submitting Eq.(h) into Eq. (9), we have

$C(x,t)=\text{C}_{\text{s}}(1-\frac{4}{\text{π}}\times\frac{\text{π}}{4})=0$ (i)

Considering Eq. (a) and (i), the Eq. (10) is proved.

$$C(x,0)=\left\{\begin{matrix} \text{C}_{\text{s}} & x=0 \\ 0 & 0<x<L \end{matrix}\right.$$

**Reference**

[A1] Abramowitz, M. and Stegun, A. I. *Handbook of Mathematical Functions with Formulas, Graphs, and Mathematical Tables (Applied Mathematics Series 55)* (ed. Abramowitz, M. and Stegun, A. I.) 87-88 (Washington U.S. Government Print. Off., 1965).

**Biographies**

Yi Liu received his PhD from Huazhong University of Science and Technology, Wuhan, in 2013. In 2013-2015, he worked as Post Doctor Fellow at the Wuhan University of Technology. He is now working at University of Toronto as a joint funding postdoctoral Fellow supported by China Postdoctoral Council and University of Toronto. His research interest is in field of optical fiber sensors for gas detection and machine tool measuring.

Yanli Li received his Bachelor degree in Mechanical and Electronic Engineering from Wuhan University of Technology in 2013. She is now a Master candidate with research interest in optical fiber sensors.

Pengcheng Huang received his Bachelor degree in School of Mechanical Science and Engineering from HuBei University of Technology in 2013. He is now a Doctor candidate with research interest in optical fiber hydrogen sensors.

Han Song received his Bachelor, Master and PhD degree in School of Mechanical Science and Engineering from Huazhong University of Science and Technology. He is now post doctor fellow at the Wuhan University of Technology with research interest in optical fiber hydrogen sensors.

Gang Zhang received his PhD from Huazhong University of Science and Technology, Wuhan, in 2000. He is now an associate professor with research interest in optical fiber gas sensors.
